# Supplementary orthopaedic screening for children and adolescents to prevent permanent skeletal deformities – protocol for the “OrthoKids” study

**DOI:** 10.1186/s12891-023-07023-3

**Published:** 2023-11-15

**Authors:** B. Scheckel, M. Naumann, D. Simic, S. Stock, O. Loose, M. Breig, K. Albrecht, K. Braun, R. Kucher, S. Deininger, L. Schmid, M. John, A. Grohnert, C. Giertz, T. Wirth

**Affiliations:** 1 Institute for Health Economics and Clinical Epidemiology (IGKE), Faculty of Medicine, University Hospital Cologne, University of Cologne, Gleueler Straße 176-178, 50935 Cologne, Germany; 2 Department of Orthopaedics, Olgahospital, Klinikum Stuttgart, Kriegsbergstraße 62, 70174 Stuttgart, Germany; 3 Association of Statutory Health Insurance Physicians Baden-Wuerttemberg (KVBW), Albstadtweg 11, 70567 Stuttgart, Germany; 4 Fraunhofer Institute for Open Communication Systems (FOKUS), Kaiserin-Augusta-Allee 31, 10589 Berlin, Germany

**Keywords:** Paediatric orthopaedics, Skeletal deformities, Screening, Children, Adolescents

## Abstract

**Background:**

Skeletal deformities (SD) in children and adolescents can lead to arthritic conditions, impairment of quality of life, and high treatment costs in the long term. However, comprehensive data on the prevalence of SDs in children and adolescents are limited and it remains therefore unclear whether there is a healthcare gap. “OrthoKids” is a project that addresses this evidence gap by implementing an orthopaedic screening for children and adolescents that supplements existing detection examinations within statutory standard care in Germany.

**Objective:**

To detect SDs so that they can be treated as needed at an early stage.

**Methods:**

The implementation of the supplementary orthopaedic screening will be evaluated through an exploratory cohort study that is set up in the German state Baden-Wuerttemberg. 20,000 children and adolescents aged 10 to 14 years will be recruited as a prospective cohort. A retrospective control cohort will be formed based on claims data provided by two cooperating statutory health insurances (SHIs). Participating children and adolescents receive a one-time orthopaedic screening. If at least one SD is diagnosed, treatment will be provided as part of the statutory standard care. Within the scope of the project, a follow-up examination will be performed after one year. An IT-platform will complement the study. The primary outcome measure is the point prevalence of scoliosis, genu varum/valgum, hip dysplasia, and flat feet. Secondary outcome measures are (i) the point prevalence of further less common SDs, (ii) health-related quality of life (HRQoL), (iii) sports ability based on activity (physical/athletic), physical constraints, and (sports) injuries, as well as (iv) monetary consequences of the orthopaedic screenings’ implementation. Implementation determinants will be evaluated, too.

**Discussion:**

If the supplementary orthopaedic screening proves to be viable, it could be considered as a supplementary examination for children and adolescents within the frame of SHI in Germany. This could relieve the burden of disease among children and adolescents with SDs. In addition, it could disburden SHIs in the medium to long term.

**Trial registration:**

The OrthoKids study was registered in the German Clinical Trials Registry (Deutsches Register Klinischer Studien (DRKS)) on 26th July 2022 under the number 00029057.

**Supplementary Information:**

The online version contains supplementary material available at https://doi.org/10.1186/s12891-023-07023-3.

## Background

Skeletal deformities (SDs) refer to congenital and acquired deviations of the bones and joints outside the norm. Between the ages of 10 and 14 the musculoskeletal system goes through a sensitive growth and transformation phase [1] during which SDs may occur, correct spontaneously, remain unchanged, or progress [2]. Adolescent scoliosis, for instance, is often associated with a risk of rapid progression during the pubertal growth spurt [3]. The higher the degree of SD in the frontal alignment of the spine (i.e., the higher the Cobb angle), the more likely progression is [4]. Such an ascending progression pattern also occurs with regard to certain SDs of the lower extremities: the further the degree of hip dysplasia develops, the greater the risk of developing degenerative arthritis of the hip and the more likely total hip replacement needs to be performed [5]. The longer O- and X-leg malposition persist outside the physiological limits, the greater the risk for degenerative arthritis of the knee and the more likely complex bone dissections need to be performed [6, 7]. Depending on the rigidity, a flat foot can increase the risk of secondary damage such as osteoarthritis (OA) [8].

While early stages of SDs usually cause little, if any, physical and psychological impact, advanced stages can have a significant impact on quality of life and sports ability, especially on the risk of injuries [9, 10]. Especially if a scoliosis has a Cobb angle > 45°, cardiopulmonary impairment and lower quality of life occur more frequently [11]. Furthermore, SDs may lead to high healthcare costs. In the United States costs of scoliosis surgery were 363 million USD in 2012 [12]. Depending on the surgical procedure, hospital costs for hip dysplasia range from 11,582 to 21,852 USD [13]. In addition, there are high treatment costs for possible complications of SDs such as OA. For example, in the United States knee OA caused 27 billion USD of healthcare expenditures [14]. In Germany, the total healthcare costs for OA were estimated to be €7,6 billion in 2008 [15].

If SDs are detected at an early stage, the use of conservative treatment measures or gentle and low-complication surgical procedures may be possible and appropriate to achieve a correction or minimisation of certain SDs. Consequently, affected children and adolescents may be spared subsequent loads of late-detected SDs such as secondary OA and subsequent arthroplasties. Depending on population, diagnostic criteria, and body region, this may also have the potential to improve health-related quality of life (HRQoL), foster sports ability and save substantial treatment costs.

Against this background, orthopaedic screening measures in children and adolescents are of particular importance. In Germany, existing early detection examinations are yet primarily focused on the prevention of general somatic diseases and mental health conditions [16]. This raises the question of whether complementary paediatric orthopaedic care should be available regarding the prevention of permanent physical limitations resulting from SDs as specific somatic conditions of the musculoskeletal system. Furthermore, whilst SDs are known to be common diagnoses in orthopaedic practice in certain service regions [17], comprehensive data on the prevalence of SDs in childhood and adolescence, their impact on HRQoL and sports ability, and their economic impact are limited.

“OrthoKids” is a project within German healthcare that addresses this evidence gap. This is done by implementing a supplementary orthopaedic screening for children and adolescents between the ages of 10 and 14 into current healthcare practice. The objective is to detect SDs so that they can be treated as needed at an early stage. The project will be supported through an IT-platform (the “OrthoKids-platform”) for data collection and management within the study process.

The evaluation of the OrthoKids project is guided by the following research questions:

1. To which extent can a supplementary orthopaedic screening support the (early) detection of SDs in the targeted population?
2. How do detected SDs affect childrens’ and adolescents’ everyday lives in terms of HRQoL and sports ability?
3. Would a supplementary orthopaedic screening entail a savings potential from the perspective of the statutory health insurance (SHI)?
4. Would a supplementary orthopaedic screening be practicable from the perspective of healthcare providers and insurants?

If the orthopaedic screening proves to be viable in a clinical, economic, and practical view, it could be considered as a supplementary early detection examination for children and adolescents within the scope of the SHI in Germany.

## Methods

This study protocol is guided by the SPIRIT 2013 checklist [18, 19]. See Additional File 1.

### Design

In the course of the OrthoKids project, an exploratory cohort study will be conducted. It is set up in the German state Baden-Wuerttemberg, i.e., a federal region that has the third-highest number of inhabitants between the ages of 10 and 14 [20]. As a prospective cohort (hereafter referred to as the “OrthoKids-cohort”), children and adolescents will be recruited for an orthopaedic screening (anticipated n = 20,000). A retrospective control group will be formed based on claims data provided by two cooperating sickness funds of the SHIs (Techniker Krankenkasse, AOK Baden-Wuerttemberg).

### Patient population

Children and adolescents are eligible for inclusion if they (i) are ≥ 10 and ≤ 14 years old, (ii) are members of any SHI, and (iii) have sufficient knowledge of the German language. Inclusion is only possible if written consent is provided by parents for participation. The consent forms and further study material is available through the website of the KVBW [21].

### Intervention

As an intervention, participating children and adolescents receive a one-time orthopaedic screening. If at least one SD is diagnosed, treatment is provided as part of statutory standard care. Progress of treatment is checked in a one-time orthopaedic follow-up examination after one year within the scope of the study.

The orthopaedic screening includes a medical history that is followed by a physical examination of the spine and lower extremities. Furthermore, information about (i) secondary orthopaedic diseases in the case of overweight, (ii) sports that endanger the skeletal system, (iii) injury prevention, and (iv) general conditions of physical and athletic ability will be provided as preventive education to both the participating children and adolescents and the parents who attend the orthopaedic appointment.

The orthopaedic screening (and eventual follow-up examination) will be delivered by orthopaedic surgeons in the ambulatory care sector who participate in outpatient medical care for persons insured under the SHI It is based on a pathway-model that was developed in accordance with specialist standards in paediatric orthopaedic care. As a decision support tool, this model serves to ensure diagnostic and therapeutic approaches according to specialist standards without disproportionately limiting individual judgment and decision making (e.g., regarding a justifiable indication for x-ray diagnostics or the appropriate format of preventive education). This specialist standard is summarised in an eLearning, in which the potentially participating orthopaedic practices take part in advance. In addition, their application interface of the OrthoKids platform will be explained there.

### Recruitment

Recruitment is carried out through a diversified marketing concept that is ought to raise awareness for the orthopaedic screening offer. This includes advertising via locally relevant print media, radio and television as well as promotion through social media. Furthermore, parents as well as children and adolescents will be specifically approached through schools, sports clubs, and fairs/festivals. In addition to these marketing strategies, the cooperating sickness funds will write to their insured in Baden-Wuerttemberg to inform them about the offer. Factual inclusion takes place through orthopaedic surgeons who provide the orthopaedic screening as an outpatient service in their practices. Children and adolescents will be recruited between August 2022 and December 2023. Potential follow-up examinations will be provided between August 2023 and December 2024.

### Outcome measures

The primary outcome measure is the point prevalence of scoliosis, hip dysplasia, genu valgum/varum, and flat foot (i.e., common SDs of the spine and lower extremities in the targeted patient population).

Secondary outcome measures are:

- Point prevalence of kyphosis, epiphyseolysis capitis femoris (ECF), and further SDs of the feet such as hallux valgus (i.e., less common SDs of the spine and lower extremities in the targeted patient population)
- HRQoL
- Sports ability as a composite of
  - Activity (physical/athletic)
  - Physical constraints
  - (Sports) injuries
- Monetary consequences of the implementation of the orthopaedic screening

In order to look at these outcomes in a differentiated way, implementation determinants will be evaluated. In the sense of Damschroder et al. [22], we define these as facilitating and impeding factors related to the provision or receipt of orthopaedic screening and the uptake of the accompanying OrthoKids-platform as a study tool.

### Sample size calculation

The sample size was determined based on data regarding the detection of scoliosis since reliable data regarding other SDs in children and adolescents between 10 and 14 is currently not available for Germany. According to the German KIGGs-survey, existing early detection measures for children and adolescents between the ages of 12 and 14 show a point prevalence of 14.8% regarding scoliosis [23]. This contrasts with a point prevalence of 10.5% without these measures [23]. Based on this, we estimate in a conservative assumption that at least 12% of included children/adolescents will be diagnosed with scoliosis in the OrthoKids cohort. Assuming an Alpha level of 0.05 and a power of 90%, this results in a required number of 7599 adolescents per group using the one-sided z-test for the comparison of two proportion values to be able to detect this difference between the two groups (10.5% vs. 12%) as significant. Since a 30% dropout rate is assumed and as the market share of the cooperating SHIs amounts to only 50% in Baden-Wuerttemberg, a sample size of 20,000 adolescents to be screened is targeted.

### Data collection

The data sources generated during data collection specifically address certain outcome measures and implementation determinants. This is depicted in Table 1.

**Table 1:** Data sources in relation to outcome measures and implementation determinants

		Data sources				
		Medical history questionnaires	Examination questionnaires	Proxy questionnaires	Claims dataset	Qualitative interviews
**Primary outcome measure**	Point prevalence of scoliosis, hip dysplasia, genu valgum/varum, and flat foot	**x**	**x**		**x**	
**Secondary outcome measures**	Point prevalence of Kyphosis, ECF, and further SDs of the feet	**x**	**x**		**x**	
	HRQoL			**x**		
	Activity (physical/athletic)			**x**		
	Physical constraints	**x**		**x**		
	(Sports) injuries			**x**		
	Monetary consequences of the implementation of the orthopaedic screening	**x**	**x**	**x**	**x**	
**Implementation determinants**	Implementation of the orthopaedic screening					**x**
	Implementation of the supporting OrthoKids-platform			**x**		**x**

### Medical history, examination, and proxy questionnaires

As shown in Table 1, three standardised questionnaires will be used.

- First, a medical history questionnaire that the attending orthopaedic surgeon will fill. It contains records of pre-existing conditions (e.g., hip dysplasia), use of orthopaedic aids (e.g., use of insoles), and physical constraints (e.g., dyspnoea under physical stress).
- Second, an examination questionnaire that attending orthopaedic surgeon will use to document findings, diagnostic recommendations, diagnoses, and treatment recommendations for the body regions to be examined.
- Third, a proxy questionnaire that will be completed by parents of participating children and adolescents. It is used to query information regarding (i) HRQoL and (ii) sports ability, i.e., a composite of activity (physical/athletic), physical constraints, and (sports) injuries.

**Fig. 1:**
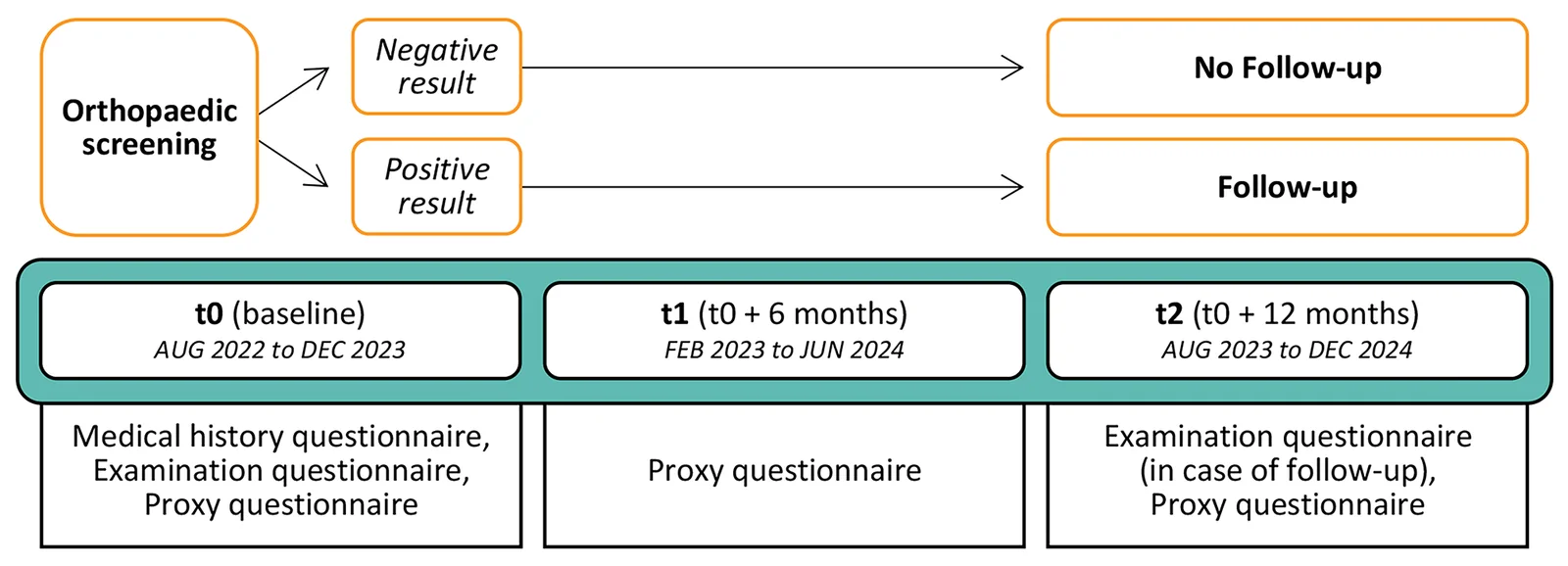
Survey via questionnaires

As Fig. 1 shows, these questionnaires will be utilised at different points of time.

The orthopaedic screening marks the baseline (*t0*). Here, initial versions of all three questionnaires are used to assess the baseline situation with respect to all primary and secondary outcomes measures. An adapted control version of the examination questionnaire will be utilised in case of a follow-up examination (*t2*). In order to check for changes in HRQoL and sports ability during the intervention, the proxy questionnaire will be sent out repeatedly, i.e., six months after the orthopaedic screening (*t1*) and at the time of the follow-up examination one year after the orthopaedic screening (*t2*). In these later versions, the usage of the “OrthoKids-App” will be briefly queried, too (i.e., a smartphone-application that presents a user component of the supporting OrthoKids-platform, see Data management).

The questionnaires regarding medical history and examination are self-designed. The proxy questionnaire, in turn, is primarily developed based on existing validated instruments.

- The Generic Score Scale of the Pediatric Quality of Life Inventory 4.0™ (PedsQL4.0)™ will be used to assess HRQoL. This instrument consists of 23 items, that are distributed over four scales each measuring another dimension (physical functioning, emotional functioning, social functioning, and school functioning). It can generally be used in children and adolescents with or without acute and/or chronic diseases and age-specific versions allow for a developmentally appropriate application [24]. The questionnaire has also been shown to be sensitive to the day-to-day problems associated with paediatric orthopaedic conditions and is often used in this conditions [25, 26].
- Physical and athletic activity will be assessed through items that originate from the MoMo-AFB. It is also an instrument with modular scales and can be used to measure a wide range of habitual physical-sport activity on behalf of different target populations [27]. It was developed and applied in course of the German Health Interview and Examination Survey for Children and Adolescents (KiGGs), that is a long-term survey to collect comprehensive health data on children and adolescents that are representative for Germany [28].
- An adapted version of a documentation form by Fuller et al. will inform items regarding physical constraints and (sports) injuries. It was originally developed for self-reporting of soccer injuries [29] and has already been used in this context in Germany [30]. The questionnaire was primarily selected because, based on data from Germany, it was assumed that most children and adolescents active in club sports play soccer [31]. Furthermore, it is increasingly used as an (adapted) instrument to query injuries in other (team) sports [32–34]. Separate items were added to the documentation form to record physical constraints that do not represent (sports) injuries (e.g., muscle cramps).
- Self-generated items will be used to query the usage of the OrthoKids-App. They concern frequency (e.g., monthly usage) and type (e.g., usage of certain functionalities).

All questionnaires were reviewed by practicing orthopaedic surgeons who were not part of the OrthoKids team to ensure their applicability. Additionally, the proxy questionnaire was tested in the orthopaedic department of the Olgahospital Stuttgart before the start of the intervention.

### Claims dataset

The two cooperating SHIs will each provide claims data at two points in time (fall 2024 and spring 2025). The first data delivery will include diagnosis data from children and adolescents in the retrospective control group to determine the point prevalence of SDs in the context of statutory standard care (without orthopaedic screening). The second data delivery will include all children and adolescents insured by the two SHIs who are diagnosed with a SD in the context of the intervention (orthopaedic screening). For this group, the data set will include diagnosis data as well as data on the utilisation of treatments such as surgeries, remedies, and aids. These data are needed to validate the health economic model of the orthopaedic screening (see Data analysis).

### Qualitative interviews

Finally, implementation determinants will be assessed via qualitative interviews with physicians or medical assistants of participating orthopaedic practices and with parents of the participating children and adolescents. In order to assure a thematic focus on implementation determinants, group-specific interview guides will be used [35]. The semi-structured interviews will be conducted during the intervention phase to account for possible changes in perspective (*t0 until end of intervention*).

### Data management

**Fig. 2:**
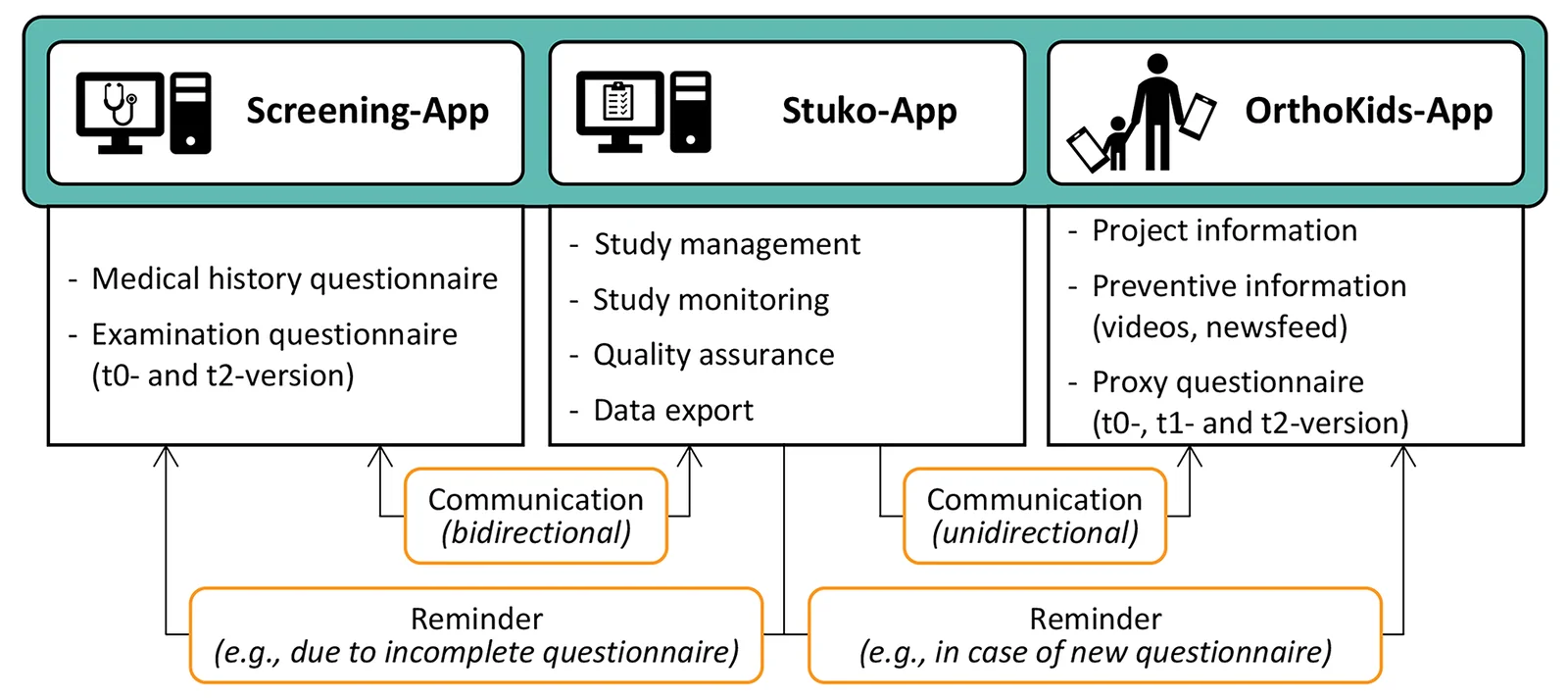
Application interfaces of the OrthoKids-platform

The orthopaedic screening will be introduced under real-world conditions. The OrthoKids-platform will facilitate the documentation process of study data. As Fig. 2 shows, it consists of three application interfaces which provide user group-specific services and functions:

- a web-application for participating orthopaedic practices (“Screening-App”)
- a web-application for study coordinators (“Stuko-App”)
- a smartphone-application for participating children and adolescents as well as their parents (“OrthoKids-App”)

The central function of the Screening-App is documentation. Findings, diagnoses, diagnostics, and treatment recommendations are documented through a standardised form (i.e., the questionnaires for medical history and examination).

The main function of the Stuko-App is coordination. This concerns multiple tasks, e.g., account creation for users of the Screening-App, first level support for users of the Screening-App and OrthoKids-App, and quality monitoring of the data collection process.

The core functions of the OrthoKids-App are information and documentation. For instance, organisational information regarding the research project can be accessed (e.g., project aims, funding, and a list of orthopaedic practices). Furthermore, a prevention offer is made available that is ought to motivate engagement in a healthy lifestyle. It consists of videos that demonstrate exercises for injury prevention and a newsfeed that provides tips from the areas of exercise, nutrition, and relaxation. Finally, the proxy questionnaire is available through this application.

Connections between these three application interfaces enable further functions. First, this includes an automated reminder system that indicates outstanding or upcoming actions in the study process. This may concern, e.g., a reminder in case of an incomplete medical history and/or examination questionnaire (Screening-App, Stuko-App) or a reminder for a new proxy questionnaire (OrthoKids-App, Stuko-App). Second, a manual communication system allows bidirectional messages between users of the Stuko-App and users of the Screening-App (i.e., participating orthopaedic surgeons can send messages to the study coordinators and vice versa through the Screening-App). Furthermore, it enables unidirectional messages from users of the Stuko-App to users of the OrthoKids-App (i.e., study coordinators can send messages to children and adolescents as well as their parents through the OrthoKids-App). These communicative functions will be inter alia utilised to distribute the proxy questionnaires and invitations to interviews, respectively.

The backend of the OrthoKids-platform contains the application logic for the coordination of study data and the described frontend functionalities. An identity- and access management system is used to administer user accounts and their permissions though a respective web-console.

In order to protect the data of all study participants, data will only be processed in a pseudonymised form. It ensures that personal identifiers that are needed for study enrolment (e.g., names or insurance numbers) and data that are needed for the evaluation of the intervention (i.e., questionnaire data, claims data, and interview data) are strictly processed according to their purpose. To uphold this purpose bound separation during the whole project, a trust centre is set up. It guarantees that only pseudonymised data are analysed.

### Data analysis

The implementation of the orthopaedic screening will be analysed through a summative evaluation concerning health-related effects (*clinical evaluation*) and cost-effectiveness (*health economic evaluation*) and through a formative evaluation concerning the implementation process (*process evaluation*). Analyses will be conducted using Excel, MAXQDA®, SPSS®, and TreeAge Pro HealthCare©.

### Clinical evaluation

In the clinical evaluation, quantitative outcome measures are statistically analysed depending on their scale level. Metric variables will be described using ranges, means, standard deviations and, if necessary, confidence intervals. For statistical testing of metric variables, the two-sided t-test is used if they are parametric. Otherwise, non-parametric tests will be used. Categorical variables are described with numbers and frequencies. In terms of statistical testing, the Chi² test is used for categorical variables. Statistical significance will be considered at a p-value ≤ 0.05. The Benjamini-Hochberg correction is applied to account for multiple testing. For missing PedsQL-data we applied the imputation algorithm proposed by Varni et al [36]. For other variables multiple imputation is applied for missing values if eligible. Depending on the research question, individual quantitative outcome measures are evaluated differently:

The primary outcome measure (i.e., the point prevalence of scoliosis, hip dysplasia, genu varum/valgum, and flat feet) will be calculated by dividing the number of each detected SDs by the overall study sample. The so-determined point prevalence of each SD in the OrthoKids-cohort will be compared to those from the retrospective control group. In addition, subgroup analyses are performed by socio-demographic (e.g., age and gender) and body-related variables (e.g., size and weight). Only confirmed diagnoses are used for these calculations. The determined point prevalence of primary SDs in the prospective cohort and in the retrospective control group are then incorporated into the health economic model. If statistically applicable, analogous analyses will be performed regarding less common SDs that are detected during the orthopaedic screening (i.e., the point prevalence of kyphosis, ECF, and further SDs of the feet as a secondary outcome measure).

Frequencies of sports injuries and physical limitations will be averaged, and a descriptive trend analysis (*t0-t1-t2*) will be performed. In addition, the mean frequencies of (sports) injuries per sports hour are calculated and considered in the trend. The timeline of the frequency of physical limitations and injuries (*t0-t2*) will provide insights into whether the education provided in the orthopaedic screening and the prevention offer provided through the OrthoKids-App have an influence on these secondary outcome measures. In addition, at t0 and t2, an unpaired t-test is used to test whether children and adolescents with deformities are more likely to suffer (sports) injuries than those without deformities.

For HRQoL-data, a descriptive comparison is first made of the total score between each SD (*t0*). Further, a before-after comparison (*t0* versus *t2*) of the mean total score per SD will be performed between each SD (paired t-test).

### Health economic evaluation

A health economic analysis will be conducted to study the mid- to long-term monetary impact of the orthopaedic screenings’ implementation. This is done based on a decision-analytic markov model in which the strategy “screening” is compared to the strategy “no screening”. The perspective is the SHI in Germany. The model aims to consider all from a cost-point-of-view relevant health states of the SDs scoliosis, hip dysplasia, genu valgum/varum, and flat feet (primary outcome measure). These and related treatments will be determined based on clinical guidelines and individual studies. In addition, the cooperating sickness funds provide claims data from their insured in the OrthoKids cohort, which are used to validate the cost data in the model. The form of analysis will be a cost-utility analysis in which the incremental cost per gained quality-adjusted life-year (QALY) is calculated. Utility values for baseline health states are calculated by transforming the PedsQL4.0™ data using validated algorithms as described by Khan et al. and Lambe et al. [37, 38], for instance. In addition, deterministic sensitivity analyses are performed for central parameters and probabilistic sensitivity analyses for all parameters [39].

### Process evaluation

A process evaluation is conducted in order to appraise the practical viability of the intervention in light of potential discrepancies between the original implementation plan and the actual implementation in the course of the OrthoKids trial [40]. The aim is to identify factors that facilitate and/or impede the orthopaedic screenings’ implementation from the perspective of participants (i.e., orthopaedic practices as well as parents of participating children and adolescents). These factors could concern the implementation of the orthopaedic screening itself (e.g., factors relating to recruitment) or the implementation of the OrthoKids-platform as an evaluative instrument that may influence the implementation of the intervention (e.g., factors relating to the functionality of the OrthoKids-platform). If the summative evaluation leads to inconclusive or negative findings, the results of the process evaluation may help to understand if this is due to inherent factors of the intervention itself or due to implementation factors [41].

Analyses regarding these implementation determinants are primarily based on verbatim transcripts of the qualitative interviews with deliverers and recipients of the intervention (see Data collection) and will be guided by a qualitative content-analytical approach that allows for interpretation of subjective meaning [42].

In addition to these qualitative analyses, descriptive-statistical analyses will be carried out to depict the usage of the smartphone-application as a potential implementation determinant more systematically. The proxy questionnaire will be utilised to collect the data required for this purpose (see Data collection).

## Discussion

The aim of the supplementary orthopaedic screening is to detect SDs so that they can be treated as needed at an early stage. The components of the orthopaedic screening are based on established diagnostic and therapeutic best practices in paediatric orthopaedic care. In contrast, the orthopaedic screening offer represents a novelty regarding the existing offer within the German SHI. It is to be expected that the evaluation of this intervention, which in this respect can be described as innovative, will provide a variety of implications for research and practice.

- The *clinical evaluation* will provide so-far missing evidence regarding the detected prevalence of common and less common SDs in children and adolescents in Germany. Thus, the results will provide insight into whether there is a healthcare gap with regard to early detection of SDs. Furthermore, through its secondary focus on HRQoL and sports ability, it will give insights into what impact these SDs may have on affected childrens’ and adolescents’ everyday lives and whether early care can achieve improvement here.
- Analyses in the course of the *health economic evaluation* will allow statements whether the early detection of SDs through an orthopaedic screening entails cost savings for the SHI in Germany. The decision-analytical modeling approach allows consideration of the effects on later-onset conditions such as OA and therefore a mid- to long-term view on the cost-effectiveness of the screening.
- The *process evaluation*, finally, provides exploratory evidence as to whether and under which conditions a supplementary orthopaedic screening may be a valuable instrument to complement existing early detection examinations for children and adolescents in Germany.

However, executing this study protocol comes with several challenges. Initially, this concerns recruitment: In order to generate a reliable database, a comparatively large sample size is needed. Recruitment in the field of health services research projects may be impeded due to several provider- and study participant-related factors [43]. In the context of OrthoKids, this e.g., could include limited screening capacities on behalf of participating orthopaedic practices or (partial) failure of strategies utilised to advertise the orthopaedic screening offer. To counter this, recruitment will be supported through cooperations with specialty societies in (paediatric) orthopaedics, local education and health authorities, and local sports clubs. In addition, the cooperation with the two sickness funds promises a regionally comprehensive approach of eligible children and adolescents. In addition to these recruitment strategies, a slight extension of the recruitment period could be considered to achieve the targeted sample size for the OrthoKids-cohort.

Furthermore, participants’ engagement with the OrthoKids-platform is crucial for the collection of study data. However, various factors on behalf of users and/or in relation to the technology itself may obstruct sufficient integration into the participants’ digital routines. This may concern, for instance, insufficient digital health literacy or insufficient system integration of applications as hindrances for digitalisation [44]. Consequently, (continuous) uptake of the web- and smartphone-application may be impeded. To encounter IT-related obstacles, paper-based versions of all questionnaires are available as backup means for data collection.

Finally, a third challenge concerns the analysis of data based on proxy-reports, since parents may over- or underestimate certain aspects compared to the children’s perspective. Thus, when assessing HRQoL, individual domains tend to be rated different by parents than by the children themselves with no clear direction discernible [45–47]. However, for the PedsQL4.0™ parent/child agreement appears to be moderate to good [48]. Since there is little literature on the proxy elicitation of sports ability and usage of smartphone-applications, its effects are difficult to assess. However, we assume little influence of the proxy elicitation at least regarding sports ability because this outcome measure mainly consists of objective items (e.g., questions regarding type, frequency, and duration of athletic activity in terms of school and club sports). Furthermore, since the analyses of the qualitative interviews with the parents are focused on their subjective experiences regarding the study participation, they may provide initial indications as to whether and to what extent a potential proxy bias may be given.

If these challenges are managed and the orthopaedic screening proves to be viable in clinical, economic, and practical view, it could be considered as a supplementary early detection examination for children and adolescents within the frame of the SHI in Germany. This could relieve the burden of disease among children and adolescents with SDs by reducing the need of surgical treatment. This may also disburden the SHI in the medium to long term.

## Electronic supplementary material

Below is the link to the electronic supplementary material.

Supplementary Material 1

## Data Availability

Not applicable.

## Abbreviations

ECF: Epiphyseolysis capitis femoris
HRQoL: Health-related Quality of Life
OA: Osteoarthritis
PedsQL4.0™: Pediatric Quality of Life Inventory 4.0™
QALY: Quality-adjusted life year
SD: Skeletal deformity
SHI: Statutory health insurance
