# Correction: Supplementary orthopaedic screening for children and adolescents to prevent permanent skeletal deformities – protocol for the “OrthoKids” study

**DOI:** 10.1186/s12891-026-10071-0

**Published:** 2026-10-06

**Authors:** B. Scheckel, M. Naumann, D. Simic, S. Stock, O. Loose, M. Breig, K. Albrecht, K. Braun, R. Kucher, S. Deininger, L. Schmid, M. John, A. Grohnert, C. Giertz, T. Wirth

**Affiliations:** 1 Institute for Health Economics and Clinical Epidemiology (IGKE), Faculty of Medicine, University Hospital Cologne, University of Cologne, Gleueler Straße 176-178, Cologne, 50935 Germany; 2 Department of Orthopaedics, Olgahospital, Klinikum Stuttgart, Kriegsbergstraße 62, Stuttgart, 70174 Germany; 3 Association of Statutory Health Insurance Physicians Baden-Wuerttemberg (KVBW), Albstadtweg 11, Stuttgart, 70567 Germany; 4 Fraunhofer Institute for Open Communication Systems (FOKUS), Kaiserin- Augusta-Allee 31, Berlin, 10589 Germany

**Correction: BMC Musculoskelet Disord 24**,** 887 (2023)**

**https://doi.org/10.1186/s12891-023-07023-3**

Following the publication of the original article [1], we found points we would like to correct.

**1.** Original sentence: “Since a 20% dropout rate is assumed and as the market share of the cooperating SHIs amounts to only 50% in Baden-Wuerttemberg, a sample size of 20,000 adolescents to be screened is targeted.”

There is a typo here. The correct version of this sentence is: “Since a 30% dropout rate is assumed and as the market share of the cooperating SHIs amounts to only 50% in Baden-Wuerttemberg, a sample size of 20,000 adolescents to be screened is targeted.”

**2.** Original sentence: “For missing values multiple imputation is applied.”

There is an analytical imprecision here. The correct version of this sentence is: “For missing PedsQL-data we applied the imputation algorithm proposed by Varni et al [36]. For other variables multiple imputation is applied for missing values if eligible.”

“36. Varni JW. Scaling and Scoring for the Acute and Standard versions of the Pediatric Quality of Life InventoryTM PedsQLTM. Version 21.3: March 2023 [Internet]. [cited 2026 Jan 14]. Available: https://www.pedsql.org/PedsQL-Scoring.pdf”.

**3.** Original sentence: “In addition, at t0 and t2, a paired t-test is used to test whether children and adolescents with deformities are more likely to suffer (sports) injuries.”

There is an analytical imprecision here. The correct version of this sentence is: “In addition, at t0 and t2, an unpaired t-test is used to test whether children and adolescents with deformities are more likely to suffer (sports) injuries than those without deformities.

**4.** Due to the addition of one citation (number 36), all following citation numberings are shifted one higher:

36. Varni JW. Scaling and Scoring for the Acute and Standard versions of the Pediatric Quality of Life InventoryTM PedsQLTM. Version 21.3: March 2023 [Internet]. [cited 2026 Jan 14]. Available: https://www.pedsql.org/PedsQL-Scoring.pdf.

37. Khan KA, Petrou S, Rivero-Arias O, Walters SJ, Boyle SE. Mapping EQ-5D utility scores from the PedsQL™ generic core scales. PharmacoEconomics. 2014;32(7):693–706.

38. Lambe T, Frew E, Ives NJ, Woolley RL, Cummins C, Brettell EA, et al. Mapping the Paediatric Quality of Life Inventory (PedsQL™) Generic Core Scales onto the Child Health Utility Index-9 dimension (CHU-9D) score for economic evaluation in children. PharmacoEconomics. 2018;36(4):451–65.

39. Briggs AH, Weinstein MC, Fenwick EAL, Karnon J, Sculpher MJ, Paltiel AD, et al. Model parameter estimation and uncertainty analysis: a report of the ISPORSMDM modeling Good Research practices Task Force Working Group-6. Med Decis Mak Int J Soc Med Decis Mak. 2012;32(5):722–32.

40. Stetler CB, Legro MW, Wallace CM, Bowman C, Guihan M, Hagedorn H, et al. The role of formative evaluation in Implementation Research and the QUERI experience. J Gen Intern Med. 2006;21(Suppl 2):1–8.

41. Proctor E, Silmere H, Raghavan R, Hovmand P, Aarons G, Bunger A, et al. Outcomes for implementation research: conceptual distinctions, measurement challenges, and research agenda. Adm Policy Ment Health. 2011;38(2):65–76.

42. Schreier M. Qualitative content analysis in practice. Los Angeles: SAGE; 2012. p. 272.

43. Krebs F, Lorenz L, Nawabi F, Lück I, Bau AM, Alayli A, et al. Recruitment in Health Services Research—A study on facilitators and barriers for the recruitment of community-based Healthcare Providers. Int J Environ Res Public Health. 2021;18(19):10,521.

44. Burmann A, Tischler M, Faßbach M, Schneitler S, Meister S. The role of Physicians in Digitalizing Health Care Provision: web-based Survey Study. JMIR Med Inform. 2021;9(11):e31527.

45. Jozefiak T, Larsson B, Wichstrøm L, Mattejat F, Ravens-Sieberer U. Quality of life as reported by school children and their parents: a cross-sectional survey. Health Qual Life Outcomes. 2008;6(1):34.

46. Kaartina S, Chin YS, Fara Wahida R, Woon FC, Hiew CC, Zalilah MS, et al. Adolescent self-report and parent proxy-report of health-related quality of life: an analysis of validity and reliability of PedsQL™ 4.0 among a sample of Malaysian adolescents and their parents. Health Qual Life Outcomes. 2015;13(1):44.

47. Papp ZK, Török S, Szentes A, Hosszú D, Kökönyei G. Parent-child agreement on health-related quality of life: the role of perceived consequences of the child’s chronic Illness. Psychol Health. 2022;0(0):1–19.

48. Varni JW, Limbers CA, Burwinkle TM. Parent proxy-report of their children’s health-related quality of life: an analysis of 13,878 parents’ reliability and validity across age subgroups using the PedsQL™ 4.0 generic core scales. Health Qual Life Outcomes. 2007;5(1):2.

The original article [1] has been updated.
